# Nine Mitochondrial Genomes of the Pyraloidea and Their Phylogenetic Implications (Lepidoptera)

**DOI:** 10.3390/insects12111039

**Published:** 2021-11-18

**Authors:** Xiaomeng Liu, Mujie Qi, Haizhen Xu, Zhipeng Wu, Lizong Hu, Mingsheng Yang, Houhun Li

**Affiliations:** 1 College of Life Science and Agronomy, Zhoukou Normal University, Zhoukou 466001, China; lxmxm_99@126.com (X.L.); xu2000949828@163.com (H.X.); wzp_0_0@163.com (Z.W.); hulizong@126.com (L.H.); 2 College of Life Sciences, Nankai University, Tianjin 300071, China; qimujie@nankai.edu.cn

**Keywords:** mitogenome, Crambidae, Pyralidae, *Orybina*, phylogeny

## Abstract

**Simple Summary:**

The Pyraloidea is a large superfamily of Lepidoptera in species composition. To date, the higher-level phylogenetic relationships in this group remain unresolved, and many taxa, with taxonomic positions historically established by morphological characters, need to be confirmed through sequencing of DNA, including mitochondrial genome sequences (mitogenomes). Here, we newly generated nine complete mitogenomes for Pyraloidea that shared identical gene content, and arrangements that are typical of Lepidoptera. The current phylogenetic results confirmed previous multilocus studies, indicating the effectiveness of mitogenomes for inference of Pyraloidea higher-level relationships. Unexpectedly, *Orybina* Snellen was robustly placed as basal to the remaining Pyralidae taxa, rather than nested in the Pyralinae of Pyralidae as morphologically defined and placed. Our results bring a greater understanding to Pyraloidea phylogeny, and highlight the necessity of sequencing more pyraloid taxa to reevaluate their phylogenetic positions.

**Abstract:**

The Pyraloidea is one of the species-rich superfamilies of Lepidoptera and contains numerous economically important pest species that cause great loss in crop production. Here, we sequenced and annotated nine complete mitogenomes for Pyraloidea, and further performed various phylogenetic analyses, to improve our understanding of mitogenomic evolution and phylogeny of this superfamily. The nine mitogenomes were circular, double-stranded molecules, with the lengths ranging from 15,214 bp to 15,422 bp, which are comparable to other reported pyraloid mitogenomes in size. Gene content and arrangement were highly conserved and are typical of Lepidoptera. Based on the hitherto most extensive mitogenomic sampling, our various resulting trees showed generally congruent topologies among pyraloid subfamilies, which are almost in accordance with previous multilocus studies, indicating the suitability of mitogenomes in inferring high-level relationships of Pyraloidea. However, nodes linking subfamilies in the “*non-PS clade*” were not completely resolved in terms of unstable topologies or low supports, and future investigations are needed with increased taxon sampling and molecular data. Unexpectedly, *Orybina* Snellen, represented in a molecular phylogenetic investigation for the first time, was robustly placed as basal to the remaining Pyralidae taxa across our analyses, rather than nested in Pyralinae of Pyralidae as morphologically defined. This novel finding highlights the need to reevaluate *Orybina* monophyly and its phylogenetic position by incorporating additional molecular and morphological evidence.

## 1. Introduction

The Pyraloidea is one of the largest superfamilies in Lepidoptera and includes more than 16,000 described extant species with a worldwide distribution except Antarctica [1,2,3,4]. The food plants of Pyraloidea are highly diverse and contain many widely cultivated crops, such as corn for *Ostrinia* spp., rice for *Chilo suppressalis*, and soybeans for *Omiodes indicate*. Thus, a number of pyraloid taxa are important pest species, which cause great losses in agricultural production [5].

A total of two families have been defined for Pyraloidea. The Crambidae comprises 63% of the pyraloid species assigned to 15 subfamilies recently defined by Léger et al. [4], and the remaining species constitute the Pyralidae, which includes five traditionally recognized subfamilies. To date, phylogenetic relationships in Pyraloidea, especially among subfamilies, remain unresolved despite extensive investigations based on morphological and genetic data [3,4,6,7,8,9,10,11,12,13,14]. In molecular investigations, a landmark study was conducted by Regier et al. [10] that provided a subfamily-level phylogenetic framework for Pyraloidea based on analyses of five nuclear genes for 42 pyraloids and a sub-dataset consisting of 19 genes for 21 of the 42 pyraloids. Recently, Léger et al. [4] proposed a thirteen-subfamily classification for Crambidae based on ten genes, and indicated that the species-rich groups in Pyraloidea, such as Acentropinae, Epipaschiinae, Pyralinae and Phycitinae, greatly need systematic revision.

The typical arthropod mitochondrial genome (mitogenome) is a circular double-stranded molecule and generally consists of 13 protein-coding genes (PCGs), two ribosomal RNA genes (rRNAs) and 22 transfer RNA genes (tRNAs) [15,16]. In addition, several noncoding elements, including the control region regulating the replication and transcription of the mitogenome, are present [17]. The mitogenome represents one kind of important molecular data used in studies on molecular evolution, phylogenetic investigation, and population genetics of insects, mainly because they are characterized by cellular abundance, absence of introns, rapid evolution, and a lack of extensive recombination [15,16]. In recent years, increasing numbers of mitogenomes have been sequenced, which in parallel has provided effective data for phylogenetic studies on multiple insect groups, such as Lepidoptera [18], Hemiptera [19,20], Coleoptera [21] and Hymenoptera [22,23].

In Pyraloidea, the mitogenomes of approximately 60 species from 12 subfamilies of two families have been sequenced to date (GenBank, August 2021). However, most of these mitogenomes were reported individually based on a simple description [24,25,26]. Zhu et al. [11] sequenced six pyraloid mitogenomes, and in combination with the other 26 available mitogenomes, performed a phylogenetic analysis for Pyraloidea. The four subfamilies in Pyralidae showed identical topology with that of Regier et al. [10], whereas the relationships among the six crambid subfamilies showed discrepancy with Regier et al. [10] and Léger et al. [4], mainly exhibited by the positions of Acentropinae and Glaphyrinae in Crambidae. More recently, Qi et al. [14] reported the first three mitogenomes for Odontiinae of Crambidae, and phylogenetic analyses of 40 pyraloid taxa confirmed the position of Odontiinae as sister to Glaphyrinae, which was also recovered by Regier et al. [10] and Léger et al. [4].

Given that the Pyraloidea is a large superfamily in species composition and many taxonomic changes at genus, tribe and even subfamily levels have been recently proposed by molecular studies [4,12,13], phylogenetic positions of more taxa or groups, historically established only by morphological or biological characters, are necessary to be confirmed through sequencing of DNA, including the mitogenomic sequences. In this study, the complete mitogenomes of nine additional pyraloid species were sequenced and annotated for the first time, and in combination with all other 60 existing mitogenomes in GenBank, phylogenetic analyses were conducted based on five datasets and three inference methods, with the aims to: (1) improve our understanding of evolutionary relationships among major groups within Pyraloidea; and (2) confirm the phylogenetic positions of the sequenced species or representative genera in Pyraloidea, because most of them have been never examined in previous molecular phylogenetic investigations.

## 2. Materials and Methods

### 2.1. Samples, DNA Extraction and Mitogenome Sequencing

Adults were collected at Mountains Yaoshan and Jigongshan of Henan Province, China, from 2019 to 2020. Identification was conducted through morphology, by blasting the standard mitochondrial *cox1* barcode to the GenBank database, or a combination thereof [27]. A total of nine species were sequenced, six of the Pyralidae, and the remaining three species belong to Crambidae. Detailed specimen information is shown in Table S1, and voucher specimens are deposited in the Biology Laboratory of Zhoukou Normal University, China.

Total genomic DNA was extracted from the thoracic tissue of a single individual using DNeasy tissue kit (Qiagen, Germany), following the manufacturer’s instructions. A total of nine libraries (one for each species) were constructed, and sequencing was conducted using an Illumina HiSeq 2500 platform with a strategy of 150 paired-ends.

### 2.2. Mitogenome Assembly and Annotation

Raw sequences were checked for quality control using the FastQC (http://www.bioinformatics.babraham.ac.uk/projects/fastqc, accessed on 16 June 2021). Clean paired reads were obtained by AdapterRemoval version 2 [28] and SOAPdenovo version. 2.01 [29]. Then, the mitogenome was assembled using the Geneious R11 [30]. The “map to reference” strategy was selected to map all cleaned reads to an “anchor” of the standard mitochondrial *cox1* barcoding sequence amplified earlier using insect primer pair Lco1490 (F) and Hco2198 (R) [31]. After iteration up to 100 times with custom sensitivity, a target sequence with high coverage was generated. The beginning and end of the target sequence were checked, and a complete mitochondrial genome was generated and circularized using MEGA X [32] to delete the overlapping sequence. The mitogenome sequence was annotated using MITOS2 webserver [33] with invertebrate genetic code, and the gene boundaries were confirmed using MEGA X [32] by aligning the new mitogenome with previously reported pyraloid mitogenomes available on GenBank. The mitogenome map of the *O. regalis*, a representative of the nine species with mitogenomes sequenced in this study, was depicted on Tutools platform (http://www.cloudtutu.com, accessed on 12 September 2021).

### 2.3. Multiple Alignment

A total of 90 mitogenomes were analyzed, which include the nine newly sequenced in the present study, 60 downloaded from GenBank for Pyraloidea and the remaining 21 from Noctuoidea, Geometroidea, Gelechioidea and Bombycoidea as outgroup sequences (Table 1). 

Among the 37 mitochondrial genes, 13 PCGs were individually aligned with the codon-based mode in TranslatorX online platform [83]. A total of two rRNAs and 22 tRNAs were independently aligned with Q-INS-i algorithm as implemented in MAFFT online platform [84]. The aligned tRNA and rRNA sequences were filtered using ClipKIT [85] to delete ambiguously aligned sites with -g 0.8 algorithm.

### 2.4. Nucleotide Composition, Substitution Saturation and Heterogeneity of Sequence Divergence

Nucleotide composition was calculated using MEGA X [32]. Strand asymmetry was calculated according to the formulas: AT-skew = [A − T]/[A + T] and GC-skew = [G − C]/[G + C] [86]. Tests of substitutional saturation were conducted with DAMBE version 5.3.74 [87,88] based on the *Iss* (i.e., index of substitutional saturation) statistic for different datasets. For this method, if *Iss* is smaller than *Iss.c* (i.e., critical *Iss*), the sequences may have experienced little substitutional saturation [89]. The heterogeneity of sequence divergences was detected by using AliGROOVE [90], with the default sliding window size and the gaps treated as ambiguous characters.

### 2.5. Phylogenetic Analyses

A total of five datasets were generated using MEGA X [32] in combination with PhyloSuite version 1.2.1 [39]: (1) PCG12: first and second codon positions of 13 PCGs; (2) PCG123: all codon positions of 13 PCGs; (3) PCG12R: first and second codon positions of 13 PCGs plus 24 RNAs; (4) PCG123R: all codon positions of 13 PCGs plus 24 RNAs; and (5) PCGAA: amino acid sequences of 13 PCGs.

Maximum likelihood (ML) analyses were conducted using IQ-TREE 2.0.4 [91] under the partitioning schemes and corresponding substitution models (Tables S2 and S3) determined by ModelFinder [92]. Branch supports (BS) were calculated using 1000 ultrafast bootstrap replicates [93]. Bayesian inference (BI) analyses were performed with MrBayes version 3.2.6 [94] with the partitioned models (Tables S4 and S5) determined by PartitionFinder version 2.1.1 [95]. A total of twelve processors were used to perform two independent runs, each with six chains (five heated and one cold) simultaneously for 500,000 to 10,000,000 generations sampled every 100 generations. Convergences were considered to be reached when the estimated sample size (ESS) value was above 200, established by Tracer version 1.7 [96], and the potential scale reduction factor (PSRF) approached 1.0 [94]. The first 25% of samples were discarded as burn-in and the remaining trees were used to calculate posterior probabilities (PP) in a 50% majority-rule consensus tree. In addition, BI analyses were also performed using PhyloBayes MPI 1.5a [97,98]. The site-heterogeneous mixture model CAT-GTR was imposed for all datasets. Each analysis involved two independent runs, and the two runs were regarded convergent satisfactorily with the maxdiff < 0.1 calculated through the “bpcomp” program implemented in PhyloBayes MPI. A consensus tree was generated with the first 1000 trees of each run as burn-in.

## 3. Results and Discussion

### 3.1. General Features of the Sequenced Mitogenomes

**Figure 1:**
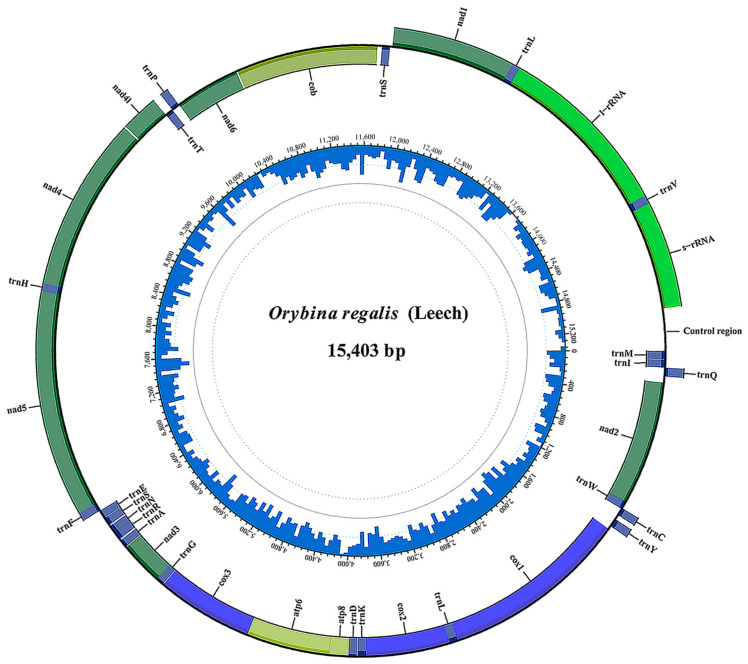
The mitochondrial genome map of *Orybina regalis*. The outer circle shows the distributions and organization of 37 mitochondrial genes, and different kinds of genes are indicated with different colors. The inner circle shows the G + C content.

A total of nine complete mitogenomes were annotated for nine species covering four subfamilies, two families of the Pyraloidea, with the lengths ranging from 15,214 bp (*S. plagialis*) to 15,422 bp (*D. rubella*) (Table 2). All mitogenomes (Figure 1) contained 37 mitochondrial genes typical in insects and showed identical gene organization to other reported pyraloid mitogenomes, which is also typical of Lepidoptera [14,16]. Similar to other insect mitogenomes [17], the A + T content of the nine mitogenomes were highly biased, showing 79.8% (*D. rubella*) to 81.7% (*S. taiwanalis*) in nucleotide composition. AT-skew and GC-skew are routinely used to describe the base composition of mitogenomes [86,99]. The negligible AT-skew and moderate GC-skew detected in the nine mitogenomes are similar to other Lepidoptera and most insect species [100]. The annotation information for the nine mitogenomes is summarized in Table S6, and all of them have been submitted to GenBank with the accession numbers shown in Table 2.

### 3.2. Tests of Substitution Saturation and Heterogeneity of Sequence Divergence

**Figure 2:**
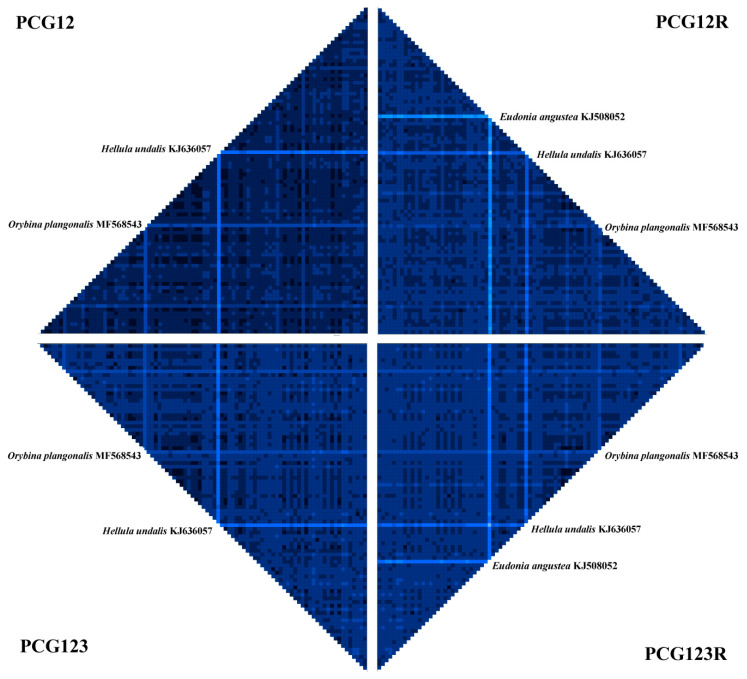
Analysis of heterogeneity of sequence divergence for four datasets. The heterogeneity of the corresponding sequence relative to other sequences increases as the indicated color becomes shallow. The species with relatively higher sequence heterogeneity are shown.

The final alignment yielded 11,211 bp of 13 combined PCGs, 2171 bp of two combined rRNAs and 1506 bp of 22 combined tRNAs. Tests of substitution saturation (Table 3) showed all observed values of *Iss* in the first and second coding positions of 13 PCGs, two rRNAs and 22 tRNA, were significantly lower than *Iss.c* values for both symmetrical and asymmetrical topologies. For the third coding positions of 13 PCGs, the value of *Iss* was significantly higher than the *Iss.c* value for asymmetrical topology, indicating some of these sites have suffered substantial saturation. Relative to the first and second coding positions of mitochondrial PCG, the third coding positions generally show a faster evolutionary rate due to synonymous mutation and might contain noise information in inferring high-level phylogenetic relationships [101]. Thus, in subsequent phylogenetic analyses, multiple datasets associated with the inclusion and exclusion of the third coding positions were considered. Analyses of sequence divergence heterogeneity (Figure 2) showed little heterogeneity among all sequences except for the *E. angustea* (KJ508052), *H. undalis* (KJ636057) and *O. plangonalis* (MF568543), which possibly ascribed to the existence of missing genes or gene fragments in these sequences.

### 3.3. Phylogenetic Analyses

By adding nine newly sequenced mitogenomes to 60 existing mitogenomes from GenBank, we performed a comprehensive phylogenetic analysis of Pyraloidea based on the hitherto most extensive mitogenome sampling, using five datasets and three inference methods.

**Figure 3:**
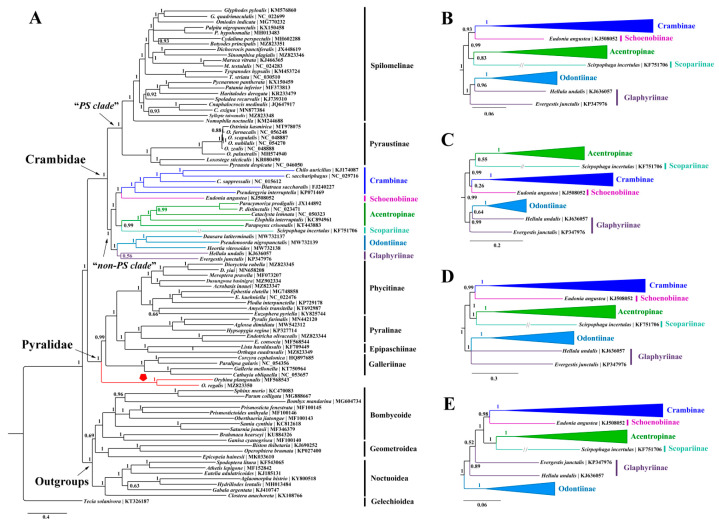
The resulting trees constructed with MrBayes for five datasets. (**A**) The whole BI tree of PCG123 dataset, and the position of the *Orybina* Snellen is emphasized with red clade and polygon; (**B**–**E**) highlight the partial BI trees (“*non-PS clade*”) constructed using the datasets of PCG12, PCG12R, PCG123R and PCGAA, respectively.

**Figure 4:**
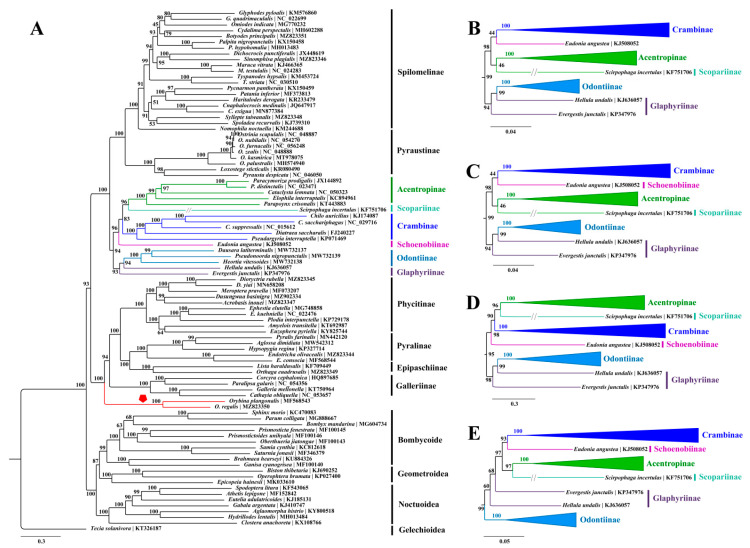
The resulting trees constructed with IQ-TREE for five datasets. (**A**) The whole ML tree of PCG123 dataset, and the position of the Orybina Snellen is emphasized with red clade and polygon; (**B**–**E**) highlight the partial ML trees (“*non-PS clade*”) constructed using the datasets of PCG12, PCG12R, PCG123R and PCGAA, respectively.

**Figure 5:**
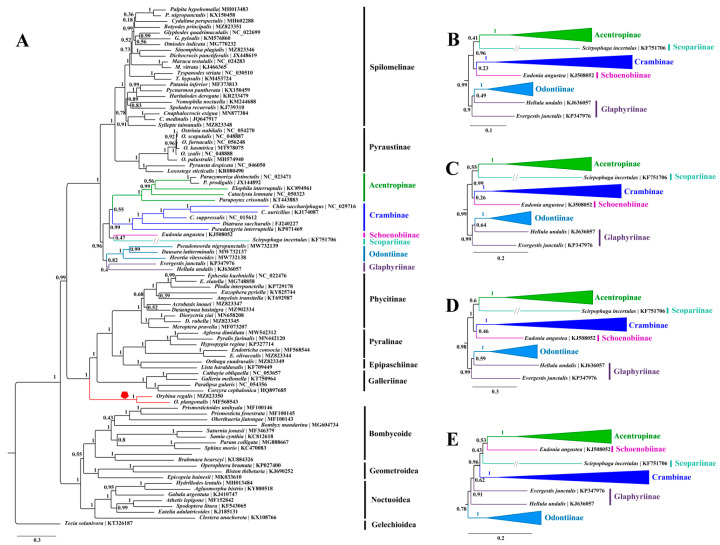
The resulting trees constructed with PhyloBayes for five datasets. (**A**) The whole BI tree of PCG123 dataset, and the position of the Orybina Snellen is emphasized with red clade and polygon; (**B**–**E**) highlight the partial BI trees (“*non-PS clade*”) constructed using the datasets of PCG12, PCG12R, PCG123R and PCGAA, respectively.

The resulting trees (Figure 3, Figure 4 and Figure 5) consistently showed the two families as monophyletic with strong supports (BS = 100, PP = 1.00) across our analyses. In Pyralidae, the relationship among four subfamilies were inferred as Galleriinae + (Phycitinae + (Pyralinae + Epipaschiinae)), which is identical with previous studies based on mitogenomic data [11,14,53,54] or multilocus data [4,10] regardless of the Chrysauginae that was not sampled herein because of the lack of an available mitogenome. Unexpectedly, *Orybina* Snellen, regarded as a member of Pyralinae morphologically [102,103], was consistently basal to the remaining taxa in the Pyralidae clade by all our analyses with high supports (BS > 90, PP > 0.90), rendering the Pyralinae paraphyletic. It should be noted that Polyterpnes, an Australian chrysaugine, was found to be basal to the Pyralidae as well [10]. *Orybina* was established in 1895, having *O. flaviplaga* from India as the type species. A total of nine species have been recorded for this genus with the distribution range generally covering the whole of Southeast Asia [102,103]. To date, molecular phylogenetic analysis of this genus has never been conducted. Zhu et al. [11] sequenced the partial mitogenome of *O. plangonalis*, but in their mitogenomic phylogenetic investigations this sequence was not sampled, probably due to its mitogenomic incompleteness. In this study, we sequenced the first complete mitogenome for *Orybina*, and conducted phylogenetic analyses with *Orybina* included for the first time. The phylogenetic position of this genus recovered herein indicated that its monophyly phylogenetic position, especially its association with the Pyralinae and Chrysauginae of Pyralidae, urgently need to be investigated, based on more extensive molecular data and morphological reassessment. In addition, the phylogenetic positions of the five other species with mitogenomes sequenced for Pyralidae were in accordance with morphological studies [1].

In Crambidae, 46 available mitogenomes (including three sequenced herein) representing eight subfamilies were sampled. All our analyses assigned the eight subfamilies into two groups, generally corresponding the “*PS clade*” and “*non-PS clade*” defined by Regier et al. [10]. The subfamilies Pyraustinae and Spilomelinae constituted the “*PS clade*” with strong support (BS ≥ 95, PP = 1.00), in accordance with previous studies based on mitogenomic data [11,14,30,33,50] or multilocus data [4,10]. In the “*non-PS clade*”, three close relationships among the six subfamilies can be recognized in most of our analyses. Of these, one was the Glaphyriinae and Odontiinae that corresponds to the “*OG clade*” in Regier et al. [10] and Léger et al. [4], although the two Glaphyriinae taxa sampled herein often did not cluster with each other, as also recovered by Qi et al. [14] using mitogenomic data. The sister relationship between Schoenobiinae and Acentropinae was recovered by all our analyses, except the PhyloBayes method of PCG123 dataset that placed Schoenobiinae as sister to Scopariinae but with weak support (PP < 0.5). The third was the close relationship between Crambinae and Scopariinae, that was also defined by Léger et al. [4] and in our analyses; only the ML method of PCGR dataset and PhyloBayes method of PCG123 dataset rejected this relationship. The “*non-PS clade*” has received intense attention in recent molecular phylogenetic investigations and several revisions have been proposed, which effectively supplemented the morphologically taxonomic systems [4,10,11,12,14]. However, nodes linking some subfamilies in the “*non-PS clade*” remain unresolved. A reason for this may be that the taxon sampling in our present study and related studies remains limited relative to this speciose group [4]. Consequently, future investigations based on increased taxon sampling and molecular data (including mitogenomic and nuclear genes or even the nuclear genome data) are needed to clarify the higher-level relationships, and to confirm or revise the groups or taxa that have never been included in previous molecular phylogenetic studies.

The three mitogenomes sequenced for Crambidae in this study were all nested into Spilomelinae in our resulting trees, reinforcing their positions in this subfamily established by morphological evidence [8]. The Spilomelinae, with 4132 species assigned to 340 genera [4], represents the most speciose subfamily in Pyraloidea. The classification of this speciose subfamily had long been regarded as inconclusive, until recent studies conducted by Mally et al. [13] and Léger et al. [4]. However, great efforts are still needed to assign the taxonomically unplaced genera or unexamined genera in molecular phylogenetic investigations to the Spilomelinae tribes [13].

## 4. Conclusions

In this study, nine complete mitogenomes were determined for Pyraloidea, and comparative mitogenomics showed these mitogenomes were conserved in nucleotide composition, gene content and gene organization in Pyraloidea and typical for Lepidoptera. Based on the hitherto most extensive mitogenomic sampling, various phylogenetic trees of five datasets and three inference methods showed the relationships among the twelve included pyraloid subfamilies, which were generally congruent and provided robust supports for previous multilocus studies, indicating the suitability of the mitogenomes for inferring higher-level relationships of the Pyraloidea. Unexpectedly, *O. regalis*, a member of Pyralinae morphologically, was consistently basal to the remaining Pyralidae taxa together with *O. plangonalis*, raising the need to reevaluate the taxonomic status of *Orybina* by incorporating molecular and morphological evidence.

## Figures and Tables

**Table 1:** The species used in phylogenetic analyses.

Superfamily	Family	Taxonomy	Species	GenBank Accession Number	Mitogenome Size (bp)	Reference
Pyraloidea	Crambidae	Spilomelinae	*Glyphodes pyloalis*	KM576860	14,960	[34]
			*G. quadrimaculalis*	NC_022699	15,255	[35]
			*Omiodes indicata*	MG770232	15,367	[36]
			*Cydalima perspectalis*	MH602288	15,232	[37]
			*Botyodes principalis*	MZ823351	15,262	This study
			*Palpita nigropunctalis*	KX150458	15,226	[38]
			*P. hypohomalia*	MH013483	15,280	[39]
			*Dichocrocis punctiferalis*	JX448619	15,355	[40]
			*Sinomphisa plagialis*	MZ823346	15,214	This study
			*Maruca vitrata*	KJ466365	15,385	Unpublished
			*M. testulalis*	NC_024283	15,110	[24]
			*Tyspanodes hypsalis*	KM453724	15,329	[41]
			*T. striata*	NC_030510	15,255	[11]
			*Pycnarmon pantherata*	KX150459	15,545	[38]
			*Patania inferior*	MF373813	15,348	[38]
			*Haritalodes derogata*	KR233479	15,253	[42]
			*Spoladea recurvalis*	KJ739310	15,273	[43]
			*Cnaphalocrocis medinalis*	JQ647917	15,368	[44]
			*C. exigua*	MN877384	15,262	[45]
			*Syllepte taiwanalis*	MZ823348	15,264	This study
			*Nomophila noctuella*	KM244688	15,309	[46]
		Pyraustinae	*Ostrinia scapulalis*	NC_048887	15,311	[47]
			*O. nubilalis*	NC_054270	14,838	[48]
			*O. zealis*	NC_048888	15,208	[47]
			*O. furnacalis*	NC_056248	15,241	[49]
			*O. kasmirica*	MT978075	15,214	[26]
			*O. palustralis*	MH574940	15,246	[25]
			*Loxostege sticticalis*	KR080490	15,218	[50]
			*Pyrausta despicata*	NC_046050	15,389	Unpublished
		Acentropinae	*Paracymoriza prodigalis*	JX144892	15,326	[51]
			*P. distinctalis*	NC_023471	15,354	[52]
			*Cataclysta lemnata*	NC_050323	15,333	Unpublished
			*Elophila interruptalis*	KC894961	15,351	[53]
			*Parapoynx crisonalis*	KT443883	15,374	[38]
		Schoenobiinae	*Scirpophaga incertulas*	KF751706	15,220	[43]
		Crambinae	*Chilo auricilius*	KJ174087	15,367	[54]
			*C. sacchariphagus*	NC_029716	15,378	Direct Submission
			*C. suppressalis*	NC_015612	15,395	[55]
			*Diatraea saccharalis*	FJ240227	15,490	[56]
			*Pseudargyria interruptella*	KP071469	15,231	[57]
		Scopariinae	*Eudonia angustea*	KJ508052	15,386	[18]
		Odontiinae	*Dausara latiterminalis*	MW732137	15,147	[14]
			*Pseudonoorda nigropunctalis*	MW732139	15,084	[14]
			*Heortia vitessoides*	MW732138	15,237	[14]
		Glaphyriinae	*Hellula undalis*	KJ636057	14,678	[58]
			*Evergestis junctalis*	KP347976	15,438	[11]
	Pyralidae	Phycitinae	*Dioryctria rubella*	MZ823345	15,422	This study
			*D. yiai*	MN658208	15,430	[59]
			*Meroptera pravella*	MF073207	15,260	[60]
			*Dusungwua basinigra*	MZ902334	15,328	This study
			*Acrobasis inouei*	MZ823347	15,239	This study
			*Ephestia elutella*	MG748858	15,346	[61]
			*E. kuehniella*	NC_022476	15,295	[62]
			*Plodia interpuncella*	KP729178	15,287	[63]
			*Amyelois transitella*	KT692987	15,205	[64]
			*Euzophera pyriella*	KY825744	15,184	[65]
		Pyralinae	*Pyralis farinalis*	MN442120	15,204	[66]
			*Aglossa dimidiata*	MW542312	15,225	Unpublished
			*Hypsopygia regina*	KP327714	15,212	[11]
			*Endotricha olivacealis*	MZ823344	15,239	This study
			*E. consocia*	MF568544	15,201	[11]
			*Orybina plangonalis*	MF568543	14,823	[11]
			*O. regalis*	MZ823350	15,403	This study
		Epipaschiinae	*Lista haraldusalis*	KF709449	15,213	[53]
			*Orthaga euadrusalis*	MZ823349	15,268	This study
		Galleriinae	*Corcyra cephalonica*	HQ897685	15,273	[67]
			*Paralipsa gularis*	NC_054356	15,280	[68]
			*Galleria mellonella*	KT750964	15,320	[69]
			*Cathayia obliquella*	NC_053657	15,408	[70]
Bombycoidea	Sphingidae	Sphinginae	*Sphinx morio*	KC470083	15,299	[71]
		Smerinthinae	*Parum colligata*	MG888667	15,288	[72]
	Saturniidae	Saturniinae	*Samia cynthia*	KC812618	15,345	[73]
			*Saturnia jonasii*	MF346379	15,261	[74]
	Endromidae		*Prismosticta fenestrata*	MF100145	15,772	Unpublished
			*Prismostictoides unihyala*	MF100146	15,355	Unpublished
	Bombycidae	Oberthuerinae	*Oberthueria jiatongae*	MF100143	15,673	Unpublished
		Bombycinae	*Bombyx mandarina*	MG604734	15,682	Unpublished
	Brahmaeidae		*Brahmaea hearseyi*	KU884326	15,442	Direct Submission
	Eupterotidae		*Ganisa cyanogrisea*	MF100140	15,250	Unpublished
Geometroidea	Geometridae	Ennominae	*Biston thibetaria*	KJ690252	15,485	[38]
		Larentiinae	*Operophtera brumata*	KP027400	15,748	[75]
	Epicopeiidae		*Epicopeia hainesii*	MK033610	15,395	[76]
Noctuoidea	Noctuidae	Amphipyrinae	*Spodoptera litura*	KF543065	15,374	[77]
		Noctuinae	*Athetis lepigone*	MF152842	15,589	[78]
	Erebidae	Euteliinae	*Eutelia adulatricoides*	KJ185131	15,360	[79]
	Nolidae	Chloephorinae	*Gabala argentata*	KJ410747	15,337	[79]
	Erebidae	Arctiinae	*Aglaomorpha histrio*	KY800518	15,472	Direct Submission
		Herminiinae	*Hydrillodes lentalis*	MH013484	15,570	[80]
	Notodontidae	Pygaerinae	*Clostera anachoreta*	KX108766	15,456	[81]
Gelechioidea	Gelechiidae	Gelechiinae	*Tecia solanivora*	KT326187	15,251	[82]

**Table 2:** Nucleotide composition of nine newly determined mitogenomes for Pyraloidea.

Species	Mitogenome Size (bp)	A%	G%	C%	T%	AT%	AT-Skew	GC-Skew
*Syllepte taiwanalis*	15,264	40.5	7.5	10.8	41.2	81.7	−0.00857	−0.18033
*Botyodes principalis*	15,262	39.8	7.8	11.5	40.9	80.7	−0.01363	−0.19171
*Sinomphisa plagialis*	15,214	40.0	7.6	11.8	40.6	80.6	−0.00744	−0.21649
*Orthaga euadrusalis*	15,268	39.3	7.9	11.9	40.9	80.2	−0.01995	−0.20202
*Endotricha olivacealis*	15,239	39.0	7.6	11.7	41.7	80.7	−0.03346	−0.21244
*Orybina regalis*	15,403	39.8	7.6	11.4	41.2	81.0	−0.01728	−0.20000
*Dioryctria rubella*	15,422	39.0	7.7	12.4	40.8	79.8	−0.02256	−0.23383
*Dusungwua basinigra*	15,328	39.4	7.9	12.1	40.6	80.0	−0.01500	−0.21000
*Acrobasis inouei*	15,239	39.3	7.7	12.0	41.0	80.3	−0.02117	−0.21827

**Table 3:** Saturation tests of different data partitions.

Data Partitions	NumOTU	*Iss*	*Iss.cSym*	*PSym*	*Iss.cAsym*	*PAsym*
PCG1s	32	0.233	0.809	0.0000	0.554	0.0000
PCG2s	32	0.145	0.809	0.0000	0.554	0.0000
PCG3s	32	0.582	0.809	0.0000	0.554	0.0036
rRNAs	32	0.439	0.793	0.0000	0.525	0.0004
tRNAs	32	0.279	0.775	0.0000	0.492	0.0000

Note: two-tailed tests were used.

## Data Availability

All mitogenome sequences generated in this study were deposited in the GenBank under accession numbers of MZ823344–NW823351, MZ902334.

## Supplementary Materials

The following are available online at https://www.mdpi.com/article/10.3390/insects12111039/s1, Table S1: Information of samples with mitogenomes sequenced in this study, Table S2: The partitioning schemes and corresponding substitution models determined by ModelFinder for the PCG123R dataset, Table S3: The partitioning schemes and corresponding substitution models determined by ModelFinder for the PCGAA dataset, Table S4: The partitioning schemes and corresponding substitution models determined by PartitionFinder for the PCG123R dataset, Table S5: The partitioning schemes and corresponding substitution models determined by PartitionFinder for the PCGAA dataset, Table S6: Annotation and comparison of mitochondrial genome organizations of nine mitogenomes sequenced in this study
