# Supplementary material for: Nine Mitochondrial Genomes of the Pyraloidea and Their Phylogenetic Implications (Lepidoptera)
Source: Insects. 2021 Nov 18;12(11):1039. doi: 10.3390/insects12111039 (PMC8623390; doi:10.3390/insects12111039)
Supplement: Supplementary file 1 [file insects-12-01039-s001.zip › Table S1 Information of samples with mitogenomes sequenced in this study.pdf]

Table S1. Information of samples with mitogenomes sequenced in this study

Family	Subfamily	Species	Specimen acc.	Locality	Longitude/latitude	Collection time
Crambidae	Spilomelinae	*Syllepte taiwanalis*	2020HNXY0152	Xinyang, Henan, China	114°03′59″E/31°49′14″N	Jul. 2020
		Botyodes principalis	2020HNLS0093	Lushan, Henan, China	112°30′56″E/33°41′00″N	Aug. 2020
		Sinomphisa plagialis	2020HNXY0137	Xinyang, Henan, China	114°03′59″E/31°49′14″N	Jul. 2020
Pyralidae	Epipaschiinae	*Orthaga euadrusalis*	2020HNLS0063	Lushan, Henan, China	112°31′20″E/33°44′19″N	Aug. 2020
	Pyralinae	*Endotricha olivacealis*	2019HNXY0003	Xinyang, Henan, China	114°03′59″E/31°49′14″N	Jul. 2019
		Orybina regalis	2020HNLS00092	Lushan, Henan, China	112°30′56″E/33°41′00″N	Aug. 2020
	Phycitinae	*Dioryctria rubella*	2020HNXY0123	Xinyang, Henan, China	114°03′59″E/31°49′14″N	Jul. 2020
		Dusungwua basinigra	2020HNXY0122	Xinyang, Henan, China	114°03′59″E/31°49′14″N	Jul. 2020
		Acrobasis inouei	2020HNXY0139	Xinyang, Henan, China	114°03′59″E/31°49′14″N	Jul. 2020
