# Supplementary material for: Nine Mitochondrial Genomes of the Pyraloidea and Their Phylogenetic Implications (Lepidoptera)
Source: Insects. 2021 Nov 18;12(11):1039. doi: 10.3390/insects12111039 (PMC8623390; doi:10.3390/insects12111039)
Supplement: Supplementary file 1 [file insects-12-01039-s001.zip › Table S2 The partitioning schemes and corresponding substitution models determined by ModelFinder for the PCG123R dataset.pdf]

Table S2. The partitioning schemes and corresponding substitution models determined by ModelFinder for the PCG123R dataset

Partitions	Models	Data partitions
P1	GTR + F + R4	c1p1, c2p1
P2	TVM + F + I + G4	c1p2
P3	TVM + F + R6	c1p3, c3p3, a6p3, n6p3, a8p3
P4	GTR + F + R5	n1p1, n4p1, n5p1, n4lp1
P5	GTR + F + R4	n1p2, n4p2, n5p2, n4lp2
P6	TIM + F + R6	n1p3, n5p3, n4lp3
P7	TVM + F + R4	c2p2, c3p2, a6p2, cbp2
P8	TPM2 + F + R5	c2p3, n3p3
P9	GTR + F + R5	n2p1, n3p1, n6p1, a8p1
P10	GTR + F + R5	n2p2, n3p2, n6p2, a8p2
P11	TIM + F + R5	n2p3
P12	GTR + F + R5	c3p1, a6p1, cbp1
P13	TPM3 + F + R5	n4p3
P14	TIM3 + F + R5	cbp3
P15	GTR + F + R6	*rrnS*, *rrnL*
P16	GTR + F + R5	tRNAs

Note: c1–c3, n1–n6, a6, a8 and cb indicate the 13 PCGs; the p1, p2 and p3 indicate the first, second and third codon positions of each PCG respectively.
