# Supplementary material for: Nine Mitochondrial Genomes of the Pyraloidea and Their Phylogenetic Implications (Lepidoptera)
Source: Insects. 2021 Nov 18;12(11):1039. doi: 10.3390/insects12111039 (PMC8623390; doi:10.3390/insects12111039)
Supplement: Supplementary file 1 [file insects-12-01039-s001.zip › Table S3 The partitioning schemes and corresponding substitution models determined by ModelFinder for the PCGAA dataset.pdf]

Table S3. The partitioning schemes and corresponding substitution models determined by ModelFinder for the PCGAA dataset

Partitions	Models	Amino acid partitions
P1	mtMet + F + R6	ATP6, ND2, ND3, ND6
P2	JTTDCMut + F + R5	ATP8
P3	mtMet + R5	COX1, COX2, COX3, CYTB
P4	mtMet + F + R6	ND1, ND4, ND4L, ND5
