# Supplementary material for: Nine Mitochondrial Genomes of the Pyraloidea and Their Phylogenetic Implications (Lepidoptera)
Source: Insects. 2021 Nov 18;12(11):1039. doi: 10.3390/insects12111039 (PMC8623390; doi:10.3390/insects12111039)
Supplement: Supplementary file 1 [file insects-12-01039-s001.zip › Table S4 The partitioning schemes and corresponding substitution models determined by PartitionFinder for the PCG123R dataset.pdf]

Table S4. The partitioning schemes and corresponding substitution models determined by PartitionFinder for the PCG123R dataset

Partitions	Models	Data partitions
P1	GTR+ I + G	c1p1, c2p1
P2	TVM + I + G	c1p2
P3	TIM + I + G	n3p3, a6p3, c1p3, cbp3, c3p3
P4	GTR + I + G	n4lp1, n1p1, n4p1, n5p1
P5	GTR + I + G	n4lp2, n1p2, n4p2, n5p2
P6	GTR + G	n1p3
P7	TVM + I + G	c2p2, a6p2, c3p2, cbp2
P8	TVM + G	c2p3, a8p3
P9	GTR + I + G	n3p1, n2p1, a8p1, n6p1
P10	TVM + I + G	n2p2, n3p2
P11	K81UF + G	n2p3
P12	GTR + I + G	c3p1, cbp1, a6p1
P13	TVM + I + G	n4p3, n4lp3, n5p3
P14	GTR + I + G	a8p2, n6p2
P15	TIM + G	n6p3
P16	GTR + I + G	*rrnS*, *rrnL*
P17	GTR + I + G	tRNAs

Note: c1–c3, n1–n6, a6, a8 and cb indicate the 13 PCGs; the p1, p2 and p3 indicate the first, second and third codon positions of each PCG respectively.
