# Supplementary material for: Nine Mitochondrial Genomes of the Pyraloidea and Their Phylogenetic Implications (Lepidoptera)
Source: Insects. 2021 Nov 18;12(11):1039. doi: 10.3390/insects12111039 (PMC8623390; doi:10.3390/insects12111039)
Supplement: Supplementary file 1 [file insects-12-01039-s001.zip › Table S5 The partitioning schemes and corresponding substitution models determined by PartitionFinder for the PCGAA dataset.pdf]

Table S5. The partitioning schemes and corresponding substitution models determined by PartitionFinder for the PCGAA dataset

Partitions	Models	Amino acid partitions
P1	mtART + I + G + F	ATP6, ND2, ND3, CYTB
P2	mtREV + I + G+ F	ATP8, ND6
P3	mtART + I + G	COX1
P4	mtART + I + G	COX2, COX3
P5	mtART + I + G + F	ND1, ND4, ND4L, ND5
